# A retrospective observational study evaluating the safety and effectiveness of commercially available automated insulin delivery systems in people with type 1 diabetes and gastroparesis

**DOI:** 10.1111/dom.70575

**Published:** 2026-02-19

**Authors:** Rachael J. L. Tan, Anusha Ahuja, Aakanksha Shrestha, Geraldine Gallen, Ben Stotesbury, Yee Cheah, Omar G. Mustafa, Martin B. Whyte, Jonathan Croos, Adam Shooshtarian, Bu' Hussain Hayee, Marietta Stadler

**Affiliations:** ^1^ Diabetes Research Department, Faculty of Life Sciences and Medicine King's College London London UK; ^2^ Department of Diabetes, King's College Hospital NHS Foundation Trust London UK; ^3^ Department of Gastroenterology King's College Hospital NHS Foundation Trust London UK

**Keywords:** automated insulin delivery, CGM, gastroparesis, hybrid closed loop, type 1 diabetes mellitus

## Abstract

**Aims:**

A retrospective observational cohort study evaluating real‐world outcomes in adults with type 1 diabetes and gastroparesis using automated hybrid closed loop insulin delivery systems (HCL).

**Materials and Methods:**

All adults with type 1 diabetes and gastroparesis attending King's College Hospital diabetes service and using continuous glucose monitoring (CGM) from 5 October 2023 to 16 January 2025 were included. Glycaemic data (HbA_1c_, CGM metrics), hypoglycaemia awareness (Gold score), diabetes distress (DDS2), and acute complications (DKA, severe hypoglycaemia) were collected from electronic health records and glucose platforms for 12 months pre‐ and post‐HCL initiation.

**Results:**

From 1827 clinic attendees, 36 adults with gastroparesis were identified, with 17 initiating HCL. HCL and non‐HCL groups had comparable baseline characteristics and metrics (HCL: Mean(SD) HbA_1c_ 70(21)mmol/mol [8.6(1.9)%]; Time in range (TIR) 36.8 (21.9) % vs. non‐HCL: HbA_1c_ 71 (25) mmol/mol [8.6(2.3)%]; TIR 37.1(19.2)%, *p* = 0.73).

Post‐HCL, HbA_1c_ median(IQR) fell to 57(52.5–65.0) mmol/mol [7.4(7.0–8.1)%] (*p* < 0.001). TIR increased to 55.7(19.9)% (*p* < 0.001), with a significant reduction in Time above range Level 2 (TAR2) [>13.9 mmol/L/>250 mg/dL](32.7% to 14.5%, *p* = 0.014). Gold score improved (2.5 to 1.5, *p* = 0.019), but DDS2 remained unchanged. No increase in DKA or severe hypoglycaemia occurred.

**Conclusions:**

Within a specialist multidisciplinary team setting, HCL systems significantly reduce HbA_1c_ without increasing risk of severe hypoglycaemia or DKA in people with type 1 diabetes and gastroparesis.

## 1 INTRODUCTION

Gastroparesis is a syndrome characterised by delayed gastric emptying in the absence of a mechanical obstruction.^1^ It is a recognised complication of diabetes mellitus with an estimated prevalence of around 5% of people with type 1 diabetes.^2^, ^3^ People with gastroparesis typically restrict their caloric intake due to nausea, bloating, upper abdominal pain and early satiety.^4^ In turn, this leads to weight loss and significant nutritional deficiencies.^5^ Unsurprisingly, patients with gastroparesis have a significantly impaired quality of life with high incidences of both anxiety and depression.^6^, ^7^

Results from the Diabetes Control and Complications Trial (DCCT) identified that improved glycaemic control reduced the incidence of gastroparesis.^8^ However, tight glycaemic control is difficult to achieve and maintain in the context of gastroparesis due to the unpredictability of food absorption and risk of hypoglycaemia.^7^, ^9^

The advancement from continuous subcutaneous insulin infusion (CSII) in open loop to the use of hybrid closed loop (HCL) systems has been transformatory for the majority of individuals living with T1D both in terms of improved glycaemic control but also quality of life.^10^, ^11^, ^12^ However, concerns have arisen around the safety of HCL within this cohort and only a few smaller case series have evaluated the use of HCL devices in people with type 1 diabetes and gastroparesis.^13^, ^14^

In this study, we present a retrospective real‐world analysis of 36 people with type 1 diabetes and gastroparesis from a clinical cohort of people accessing a tertiary multidisciplinary outpatient diabetes service. We compare and explore the safety and benefits of using commercially available HCL therapy within this group.

## 2 MATERIALS AND METHODS

### 2.1 Study design and cohort

This was a retrospective clinical cohort study using routine clinical data following the STROBE checklist. Electronic health care records (EHRs) were used to identify all people with type 1 diabetes who attended the King's College Hospital NHS Foundation Trust (KCH) diabetes outpatient department between 5 October 2023 and 16 January 2025. Data were anonymised according to operative standards at the time of data extraction. We aimed to evaluate whether HCL systems are safe in people with type 1 diabetes and gastroparesis and whether they improve glycaemic control by decreasing HbA_1c_ and rates of hypoglycaemia.

All individuals being considered for HCL systems are discussed within the type 1 diabetes multidisciplinary team (MDT) meeting. This includes a review of their eligibility as per UK NICE guidance (TA151 and TA943) and the individual's ability and safety to operate the system.^15^, ^16^ Onboarding to HCL includes two group education sessions facilitated by diabetes educators and supported by dieticians and psychologists. Early follow up is frequent, with 2–3 contacts in the first week and weekly thereafter for up to 6 weeks. More complex individuals may be onboarded in one‐to‐one sessions with a senior diabetes educator, allowing high intensity, bespoke support and management.

People were excluded from analysis if they were not coded within the EHRs as having gastroparesis or currently using CGM with at least 70% sensor wear over a 14‐day period. Accuracy of diagnosis of type 1 diabetes and gastroparesis was checked by two independent individuals. Diagnosis of gastroparesis was taken as delayed emptying, demonstrated on a scintigraphy gastric emptying study (GES) or from documentation in the EHR that the patient clinically had gastroparesis. Two cohorts of individuals were identified: those with type 1 diabetes and gastroparesis initiated on HCL therapy (referred to as the HCL group) and those who remained on either multidose injection therapy (MDI) or open loop continuous insulin infusion (referred to as the non‐HCL group).

People within the HCL group had CGM data collected from EHRs and from glucose management platforms (Medtronic Carelink, Libre Careview, Dexcom Clarity and Glooko) at two time points: within 12 months prior to starting HCL (baseline) and within 12 months post HCL initiation. People were excluded from analysis if CGM data were not available for both time points. For people within the non‐HCL group, only the most recently available data were collected.

### 2.2 Variables

We collected the following baseline variables (age, self‐reported gender, date of diabetes diagnosis, date of gastroparesis diagnosis, ethnicity, body mass index (BMI)), glycated Haemoglobin A_1c_ (HbA_1c_) and 14 day sensor glucometrics (Time in range (TIR) 3.9–10 mmol/L or 70–180 mg/dL, Time above range Level 1 (TAR1) 10.1–13.9 mmol/L or 181–250 mg/dL, Time above range Level 2 (TAR2) >13.9 mmol/L or >250 mg/dL, Time below range Level 1 (TBR1) 3.0–3.9 mmol/L or 54–69 mg/dL, Time below range Level 2 (TBR2): <3.0 mmol/L or <54 mg/dL), % co‐efficient of variation (%CV), average blood glucose, glucose management indicator (GMI), % sensor use, total daily dose of insulin (TDD), total daily announced carbohydrate intake, number of carbohydrate entries per day, modality of diabetes treatment (MDI vs. CSII), technology choice (CGM brand, HCL brand), acute (episodes of DKA or severe hypoglycaemia in the previous 12 months) and chronic complications of diabetes (retinopathy, neuropathy and nephropathy), Gold score to assess hypoglycaemia awareness and two item diabetes distress screening score (DDS2).^17^, ^18^

We collected and compared glycaemic measures before and after HCL initiation, including HbA_1c_ and 14‐day sensor glucometrics (TIR, TBR1, TBR2, TAR1, TAR2), DDS2, Gold score and acute annualised diabetes complications.

Only people with >70% CGM sensor usage were included in the study. Where individuals were using flash CGM prior to HCL initiation, glucometrics such as TIR were calculated manually by analysing the percentage of all recorded glucose levels over a 14‐day period.

### 2.3 Statistical methods

All statistical analysis was performed using SPSS version 31.0 (IBM). Continuous variables are presented as mean and standard deviation (SD) or median and interquartile range (IQR) depending on normality of distribution as determined by Shapiro–Wilk test and Kolmogorov–Smirnov testing. Paired student *t*‐tests were performed for parametric variables (e.g., TIR and BMI) and Wilcoxon rank tests for non‐parametric variables (e.g., HbA_1c_ and TDD of insulin). Frequency comparisons were conducted using Chi‐Squared tests, with Fisher's exact test applied in situations where sample sizes were <5. A two‐sided *p* value <0.05 was considered statistically significant.

## 3 RESULTS

**FIGURE 1:**
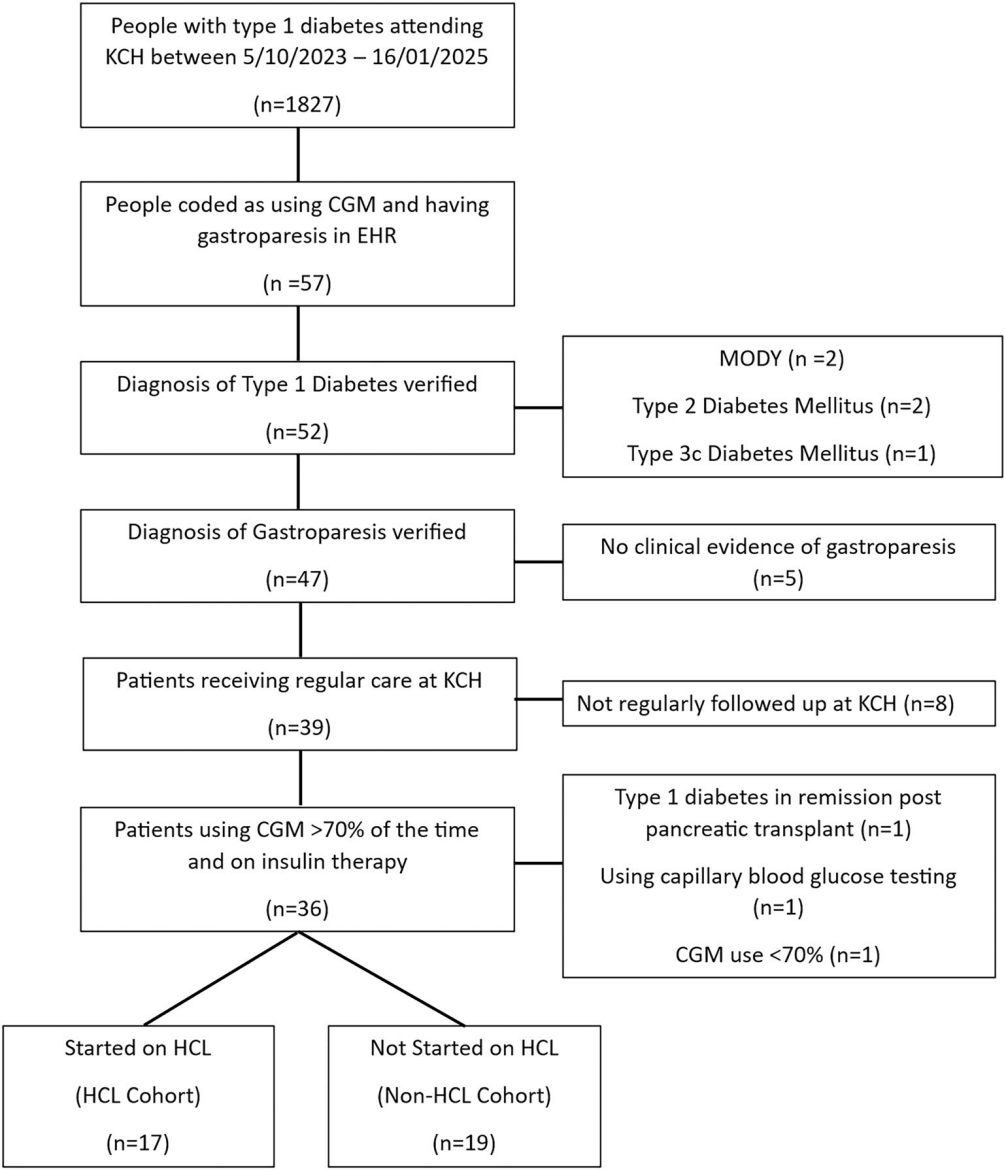
Study flow chart showing inclusion and exclusion criteria. CGM, continuous glucose monitoring; EHR, electronic health care record; HCL, Hybrid Closed Loop; KCH, King's College Hospital; MODY, Maturity Onset Diabetes of the Young.

Review of Electronic health records (EHR) identified 1827 people with type 1 diabetes who attended outpatient diabetes clinics at KCH between 5 October 2023 and 16 January 2025. Of these, 57 were coded as having gastroparesis and concurrently using CGM. Ten patients were then excluded as it was determined that the diagnosis of both type 1 diabetes and gastroparesis was not certain. Eight patients were excluded as their regular care had transferred out of area, leading to limited CGM data. A final further three patients were excluded: one as not currently taking insulin due to receiving a pancreatic transplant, one who was using capillary blood glucose testing rather than CGM, and one due to <70% CGM use. This left 36 patients for review of whom 17 were initiated on a HCL insulin pump (HCL group) and 19 who remained on either MDI or open loop CSII (non‐HCL group) (Figure 1). Of the 36 individuals within this study, gastric emptying study (GES) results were available for 21 individuals. For the remaining 15 individuals, a diagnosis of gastroparesis relied on comments within their notes that the GES was abnormal, or that clinically they were deemed to have gastroparesis.

### 3.1 HCL Group

The mean (SD) age of individuals within the HCL group was 46.3 (14.4) years with 88% being female and the majority (59%) being of white British ethnicity. Mean duration of diabetes was 33.1 (13.2) years with mean duration of gastroparesis 10.5 (8) years. Fifteen were using CSII in open loop prior to HCL initiation with two being managed on MDI therapy. The mean duration of CSII use prior to HCL initiation was 13.2 (8.9) years. There were high incidences of diabetes‐related complications with 94% having retinopathy, 71% having neuropathy, and 35.3% having CKD stage ≥3a (Table 1).

**TABLE 1:** Baseline characteristics of both the HCL and Non‐HCL groups.

Variable	HCL (*n* = 17)	Non‐HCL (*n* = 19)	*p* value
Mean (SD) unless otherwise specified			
Age, years	46.3 (14.4)	48.2 (13.7)	0.686
Sex: *number* (%)			
Female	15 (88.2)	15 (79)	0.662
Male	2 (11.8)	4 (21)	
Ethnicity: *number* (%)			
White, British/English	10 (58.8)	14 (73.7)	0.141
White, Irish	0 (0)	1 (5.3)	
Black/Black British	1 (5.9)	3 (15.7)	
Asian/ Asian British	1 (5.9)	1 (5.3)	
Mixed	1 (5.9)	0 (0)	
Any Other Ethnic Group	1 (5.9)	0 (0)	
Not stated/Undefined	3 (17.6)	0 (0)	
BMI,^a^ kg/m^2^	22.4 (19.7–25.2)	22.8 (20.8–26.8)	0.379
HbA_1c_, mmol/mol	70 (21)	71 (25)	0.731
HbA_1c_, %	8.6 (1.9)	8.6 (2.3)	0.731
% CGM wear^b^	89.5 (21.8)	94.0 (13.0)	0.138
% Time above Range Level 2 (TAR2)	32.7 (24.7)	34.6 (20.6)	0.800
% Time above Range Level 1 (TAR1)	26.1 (12.7)	26.5 (8)	0.906
% Time in Range (TIR)	36.8 (21.9)	37.1 (19.2)	0.967
% Time Below Range Level 1 (TBR1)^a^	1 (0–4)	1 (1–2)	0.754
% Time Below Range Level 2 (TBR2)^a^	0 (0–0.5)	0 (0–1)	0.975
Total Daily Insulin Dose (TDD), units^a^	19.9 (17.1–38.5)	–	
Diabetes Duration, *years*	33.1 (13.2)	31.0 (14.9)	0.686
Gastroparesis Duration, *years*	10.5 (8.0)	8.5 (6.0)	0.398
Diabetes to Gastroparesis Diagnosis, *years*	22.2 (10.5)	22.1 (13.3)	0.896
Treatment Modality: *number* (%)			
Multiple Daily Injections	2 (11.8)	16 (84)	<0.001
CSII (Open Loop)	15 (88.2)	3 (16)	<0.001
CSII Duration, years	13.2 (8.9)	11.6 (1.7)	1.000
HCL Duration, years	2.2 (1.6)	‐	
Type of CGM: *number* (%)			
Freestyle Libre 1	9 (53)	0 (0)	<0.001
Freestyle Libre 2	7 (41)	6 (31.6)	0.730
Freestyle Libre 2+	0 (0)	5 (26.3)	0.050
Freestyle Libre 3	0 (0)	1 (5.3)	1.000
Dexcom G6	1 (6)	5 (26.3)	0.050
Dexcom G7	0 (0)	1 (5.3)	1.000
Medtronic Guardian 4	0 (0)	1 (5.3)	1.000
Complications			
Diabetic Retinopathy: *number* (%)	16 (94)	16 (84.2)	0.234
Peripheral Neuropathy: *number* (%)	12 (70.6)	14 (73.7)	1.000
CKD: *number* (%)			
Stage 1	7 (41.2)	8 (42.1)	1.000
Stage 2	4 (23.5)	3 (15.8)	0.684
Stage 3a	1 (5.9)	1 (5.3)	1.000
Stage 3b	2 (11.8)	0 (0)	0.214
Stage 4	3 (17.6)	2 (10.6)	0.650
Stage 5	0 (0)	5 (26.3)	0.047
Diabetes Emergencies in previous 12 months			
DKA: *number of people*	4^c^	6^d^	0.717
Severe Hypoglycaemia: *number*	1^e^	3^f^	0.601
Gold Score^g^	2.5 (4)	3 (4)	0.854
DDS2^h^	3 (2)	3.5 (1)	0.210

*Note*: Paired student *t*‐test were performed for parametric variables and Wilcoxon rank test for non‐parametric variables. Frequency comparisons were conducted using Chi‐Square tests, with Fisher's exact test in situations with sample size <5.

Abbreviations: BMI, Body mass index; CKD, Chronic Kidney Disease; CSII, continuous subcutaneous insulin infusion; DDS2, Diabetes Distress Score 2 point; DKA, Diabetic Ketoacidosis; HCL, hybrid closed loop; IQR, interquartile range; SD, standard deviation; TIR, Time in Range; TAR1, Time Above Range Level 1; TAR2, Time Above Range Level 2; TBR1, Time below range Level 1; TBR2, Time below range level 2; TDD, total daily dose of insulin.

^a^ Non‐parametric data displayed as median (IQR).

^b^ *N* = 10 HCL with 7 individuals were using flash CGM prior to HCL. % glucometrics were calculated manually using all recorded glucose levels over a 14‐day period.

^c^ 4 individuals had 1 episode of DKA in the 12 months prior to HCL initiation.

^d^ 4 people had 1 episode of DKA, 1 per had 2 episodes and 1 person had 4 episodes of DKA within the last 12 months.

^e^ 1 person had 2 severe hypos in the 12 months prior to HCL initiation.

^f^ 1 person had 1 severe hypoglycaemic event, 1 person had 2 and 1 person had 6 severe hypoglycaemic events.

^g^ Gold score *n* = 16 for HCL and *n* = 7 for non‐HCL.

^h^ DDS2 *n* = 9 for HCL and *n* = 14 for non‐HCL.

Patients were onboarded to five different brands of commercially available HCL, selected by patient choice. The Medtronic MiniMed™ 780G with SmartGuard was chosen by 47.1%, Tandem t:slim X2 with Control‐IQ™ 23.5%, Omnipod 5 with SmartAdjust™ 17.6%, and Dana or Ypsomed pumps with CamAPS FX 11.8% (Table 2). Mean duration of follow up, post HCL initiation, was 2.2 (1.6) years (Table 1). Only three of the 17 individuals remained on the same brand of CGM after HCL initiation (two on Freestyle Libre® and one on Dexcom®). The remaining 14 switched brand of CGM to facilitate HCL functionality with their chosen HCL system.

**TABLE 2:** HCL group insulin administration at baseline and post‐HCL initiation.

Insulin administration	*n* (%)
*Insulin Delivery pre‐HCL* (*Baseline*)	
MDI	2 (11.8)
CSII (Open Loop)	15 (88.2)
Pump Model	
Medtronic MiniMed™ Paradigm Veo	1 (6)
Medtronic MiniMed™ 780G	2 (11.8)
Medtronic MiniMed™ 670G	2 (11.8)
Medtronic MiniMed™ 640G	7 (41.2)
Tandem t:slim X 2	1 (6)
Omnipod Dash	2 (13.3)
*HCL system used during follow‐up* (*post‐HCL*)	
Medtronic MiniMed™ 780G (SmartGuard)	8 (47.1)
Tandem t:slim X 2 (Control I‐Q™)	4 (23.5)
Omnipod 5 Smart Adjust™	3 (17.6)
Dana CamAPS FX	1 (5.9)
Ypsomed CamAPS FX	1 (5.9)

Abbreviations: CSII, continuous subcutaneous insulin infusion; HCL, hybrid closed loop; MDI, multiple dose injection therapy; SD, standard deviation.

### 3.2 Non‐HCL group

There were 19 individuals with type 1 diabetes and gastroparesis identified during this study that were not initiated on HCL therapy. Three of the 19 individuals were using CSII in open loop (mean duration of 11.6 (1.73) years), having declined HCL upgrades, preferring to remain in open loop (Table 1). All three of these individuals expressed concerns about loss of control and lack of trust with a HCL system. Of the 16 on MDI, 15 are eligible for HCL therapy under NICE guideline TA151 based on their HbA_1c_.^15^ Out of the 15 individuals eligible for CSII, nine have not been offered HCL currently following an MDT discussion highlighting either inability to cope with the transition to HCL or concerns about their ability to manage a HCL system safely. These nine included two individuals with dexterity issues, two individuals with impaired vision, three individuals with recurrent episodes of DKA, one individual with limited engagement with diabetes services and one individual who had significant concurrent mental health issues impairing their ability to self‐manage their diabetes. Of the remaining six individuals on MDI who have been offered HCL by the MDT, three are awaiting onboarding and three have declined, preferring to stay on MDI therapy although the reason for this was not recorded within the medical notes.

The 19 individuals within the non‐HCL group had very similar baseline demographics to the HCL group in terms of age (mean (SD) 48.2 (13.7) years), diabetes duration (31.0 (14.9) years), gender (79% females) and ethnicity (79% white British). Duration of gastroparesis was shorter but not statistically different from the HCL group (8.5 (6) years vs. 10.5 (8) years *p* = 0.398). They had a higher incidence of both severe hypoglycaemia and DKA in the previous 12 months, but neither was statistically significant (three people in the non‐HCL group compared to one in HCL *p* = 0.601, six people with DKAs in the non‐HCL group compared to four in the HCL group *p* = 0.717). Individuals within the non‐HCL group had more advanced chronic kidney disease (42.2% with CKD stage ≥3a) (Table 1).

### 3.3 Comparison between HCL and non‐HCL

**FIGURE 2:**
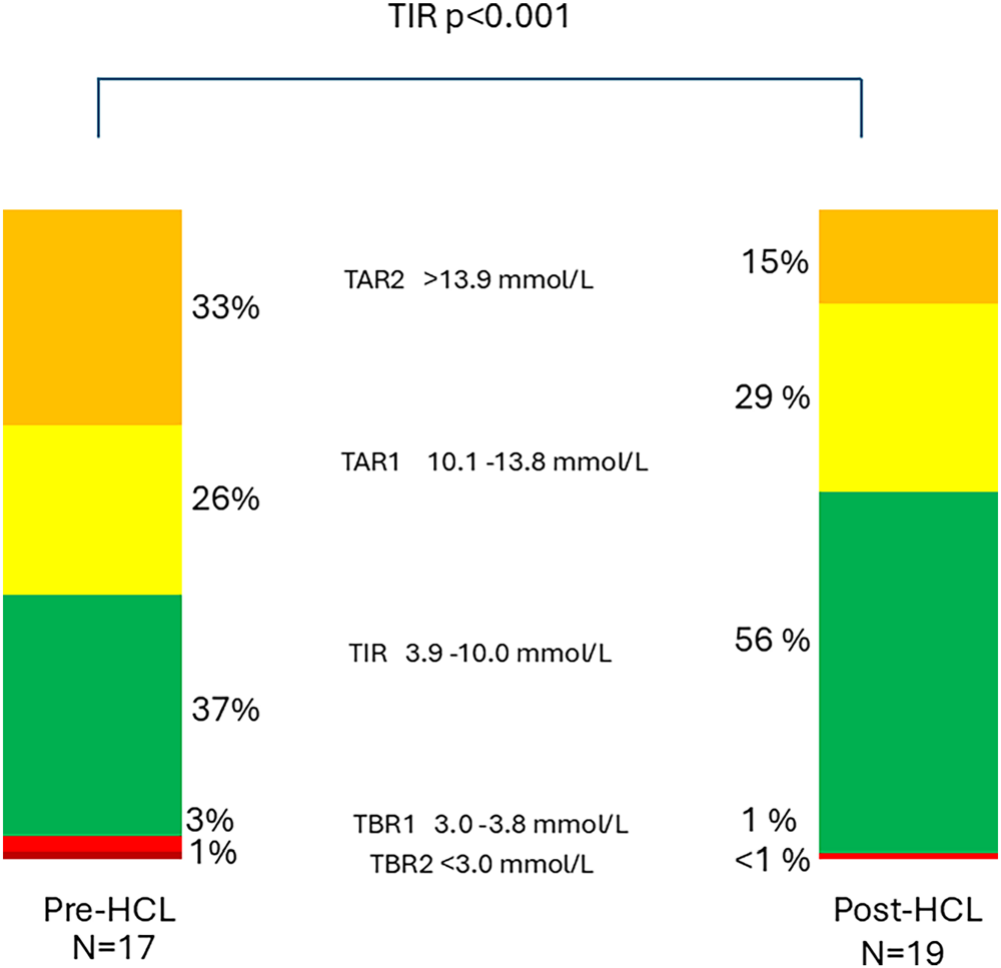
Continuous Glucose Monitoring (CGM) Time in range comparison pre and post HCL initiation. All percentages represent means. Paired t‐test comparing pre‐HCL and post‐HCL initiation % Time in Range from 14 day CGM downloads. HCL, Hybrid Closed Loop; TAR1, Time above range level 1; TAR2, Time above range Level 2; TBR1, Time below range level 1; TBR2, Time below range level 2; TIR, Time in Range.

Individuals initiated on HCL showed a drop in median (IQR) HbA_1c_ from 70 (64.5–85.5) mmol/mol or 8.6 (8.1–10.0)% to 57 (52.5–65.0) mmol/mol or 7.4 (7.0–8.1)%, (*p* < 0.001) (Table 3). There was improvement in sensor glucometrics with an increase in mean (SD) TIR increasing from 36.8 (21.9)% to 55.7 (19.9)% (*p* < 0.001) and a reduction in TAR level 2 (TAR2) from 32.7 (24.7)% to 14.5 (12.6)% (*p* = 0.014) (Figure 2). Gold score improved from a baseline median score of 2.5 (1–4.8) to 1.5 (1.0–2.8) (*p* = 0.019). There were no episodes of either DKA or severe hypoglycaemia in the 12 months post HCL start. As well as improvement in HbA_1c_, there was significant improvement in glycaemic variability from a CV% of 42.4 (4.6)% to 32.6 (7.3)% (*p* = 0.014) (Table 3).

**TABLE 3:** Comparison of glucometrics and diabetes complications pre and post HCL initiation.

Variable	Timepoint	*N*	Mean (SD) or Median^a^ (IQR)	Baseline vs. Post‐HCL (Paired test, McNear Test or Wilcoxen Signed Rank test *p*‐value)	
				MD (95%CI) or SE^a^	*p* value
TDD Insulin (units)^a^	Baseline	10	19.9 (17.1–38.5)	9.8	0.240
	Post‐HCL	17	28.7 (19.2–36.7)		
Daily announced Carb intake (g)^a^	Baseline	8	49 (23.5–99.0)	7.1	0.070
	Post‐HCL	16	70.5 (33.2–137.4)		
No of Carb entries/day^a^	Baseline	4	1.6 (1.1–3.2)	2.7	0.144
	Post‐HCL	16	3.1 (1.6–6.0)		
BMI (kg/m^2^)	Baseline	17	22.7 (3.3)	0.1 (− 0.6–0.9)	0.670
	Post‐ HCL	17	22.6 (2.9)		
HbA_1c_ (mmol/mol)^a^	Baseline	17	70 (64.5–85.5)	21.1	<0.001
	Post‐HCL	16	57 (52.5–65.0)		
HbA_1c_ (% DCCT units)^a^	Baseline	17	8.6 (8.1–10.0)	21.1	<0.001
	Post‐HCL	16	7.4 (7.0–8.1)		
Sensor Wear (%)	Baseline^b^	10	85.5 (10.7)	0 (−0.3–0.3)	1.000
	Post‐HCL	17	92.1 (10.1)		
GMI (mmol/mol)	Baseline	8	69.8 (15.8)	5.1 (−3.5–21.5)	0.128
	Post‐HCL	14	59.6 (6.8)		
Average glucose (mmol/L)	Baseline	13	12.1 (3.4)	0.6 (0.9–3.3)	0.005
	Post‐HCL	16	10.2 (1.5)		
TAR2 (%) >13.9 mmol/L >250 mg/dL	Baseline	17	32.7 (24.7)	18.2 (7.9–28.6)	0.014
	Post‐HCL	17	14.5 (12.6)		
TAR1 (%) 10.1–13.8 mmol/L 181–250 mg/dL	Baseline	17	26.1 (12.7)	−2.9 (−11.2–5.3)	0.949
	Post‐HCL	17	29 (10)		
TIR (%) 3.9–10.0 mmol/L 70–180 mg/dL	Baseline	17	36.8 (21.9)	−18.9 (−26.2 to −11.5)	<0.001
	Post‐HCL	17	55.7 (19.9)		
TBR1 (%)^a^ 3.0–3.9 mmol/L 54–69 mg/dL	Baseline	17	1 (0–4)	9.7	0.081
	Post‐HCL	17	1 (0–1.5)		
TBR2 (%)^a^ <3.0 mmol/L < 54 mg/dL	Baseline	17	0 (0–0.5)	3.7	0.104
	Post‐HCL	17	0 (0–0)		
CV (%)	Baseline	8	42.4 (4.6)	8.0 (2.2–13.8)	0.014
	Post‐HCL	16	32.6 (7.3)		
DKA^c^ *Number of people* (%)	Baseline	17	4 (23.5)	0.8	0.375
	Post‐HCL	15	0 (0)		
Severe hypoglycaemia^d^ *Number of people* (%)	Baseline	17	1 (5.9)	1.0	1.000
	Post‐HCL	15	0 (0)		
Gold Score^a^	Baseline	16	2.5 (1–4.8)	7.1	0.019
	Post‐HCL	12	1.5 (1.0–2.8)		
DDS2^a^	Baseline	9	3 (2.3–4.3)	2.7	0.063
	Post‐HCL	7	2 (1.5–2.5)		

*Note*: Parametric variables are reported as mean (±SD) and paired *t* test results reported as mean difference (MD) and 95% Confidence intervals. McNemar test performed for DKA and severe hypoglycaemia. Wilcoxon Signed Rank test used for Gold score and DDS2.

Abbreviations: BM, blood glucose; CV, co‐efficient of variation; DDS2, two item diabetes distress screening score; DKA, Diabetic Ketoacidosis; GMI, glucose management indicator; TAR1, Time above range level 1; TAR2, Time above range level 2; TBR1, Time below range level 1; TBR2, Time below range level 2; TDD, total daily dose of insulin; TIR, time in range (3.9–10 mmol/L or 70–190 mg/dL); SE, standard error.

^a^ Non‐parametric data which is reported as median (IQR) + standard error.

^b^ 7 people were on flash CGM at baseline.

^c^ 4 people had 1 episode each of DKA in the 12 months prior to starting HCL.

^d^ 1 person had 2 severe hypoglycaemic episodes in the 12 months prior to HCL initiation.

Despite a decrease in HBA_1c_ and an increase in TIR, total daily insulin dose (TDD) failed to reach a statistical difference although increased from a median (IQR) of 19 (17.2–38.5) units/day to 28.7 (19.2–36.7) *p* = 0.24 (Table 3). Similarly, there was a trend towards an increase in the total daily announced carbohydrate intake recorded from a median (IQR) of 49 (23.5–99.0) g/day to 70.5 (33.2–137.4) g/day although this narrowly missed statistical significance (*p* = 0.07).

## 4 DISCUSSION

To our knowledge, this is the largest real‐world case series of individuals with type 1 diabetes and gastroparesis who have been managed with commercially available HCL insulin systems. This retrospective clinical cohort study demonstrates that within a tertiary MDT setting, HCL safely improves glycaemia and reduces episodes of diabetic emergencies such as DKA and severe hypoglycaemia within this clinically complex cohort of individuals.

The algorithms critical to the functioning of HCL systems were not specifically designed for individuals with gastroparesis. This raises concerns about their ability to manage the marked glycaemic variability commonly observed in people with T1D and gastroparesis, with potential risks of both significant hyperglycaemia and risk of diabetic ketoacidosis (DKA) as well as an increased risk of severe hypoglycaemia.

In 2019 Kaur et al. were the first to publish a case series exploring the safety of the Medtronic 670G HCL system within this cohort.^14^ They compared five adults with type 1 diabetes and gastroparesis to nine age, sex and diabetes duration matched people with type 1 diabetes without gastroparesis. They demonstrated the safety of the HCL system and a similar reduction in HbA_1c_ and time in range (TIR) between the two groups, indicating similar efficacy in glucose control. More recently, Daly et al. have published a case series of seven people (six using Metronic Minimed™ 670G and one on CamAPS FX) with type 1 diabetes and gastroparesis showing reduction in HbA_1c_ and improved TIR.^13^

Our larger study includes 36 individuals who had a similar mean age, diabetes duration, and duration of gastroparesis to these previous studies.^13^, ^14^ Equally, we had mainly females (88%), and the majority had evidence of other microvascular complications such as retinopathy. Starting HbA_1c_ was lower within our cohort (70 (21)mmol/mol) compared to the Daly et al. cohort (83 (9) mmol/mol), but despite this, we still saw a significant decrease in HbA_1c_, improved time in range (TIR), and reduced time at level 2 hyperglycaemia (TAR2).^13^ Importantly, a reduction in glycaemic variability was demonstrated, which has been identified as a key variable to target in conjunction with HbA_1c_ in efforts to minimise microvascular complications.^19^, ^20^

Our study is the first to look at rates of diabetes complications such as DKA and severe hypoglycaemia (SH) within individuals with gastroparesis initiated on HCL therapy. At baseline, the incidence of diabetes‐related emergencies was higher in the non‐HCL group, with six people having DKA and three having severe hypoglycaemia, compared with four people having DKA and one person having severe hypoglycaemia in the HCL cohort. While individuals with severe hypoglycaemia are considered for CSII and HCL therapy under NICE guideline TA943, each individual case is assessed by the diabetes MDT to determine the ability and safety of the individual to manage an HCL system.^16^ The higher rates of diabetic emergencies in the group not onboarded to HCL likely reflect the greater proportion of individuals whom the MDT deemed not yet safe to manage a HCL system. Notably, however, the one individual within the HCL cohort who had two severe hypoglycaemic episodes prior to HCL initiation has had none since onboarding to HCL despite nearly 2.5 years of follow up. These data corroborate previous publications indicating that HCL systems are safe in individuals with type 1 diabetes and gastroparesis.^13^, ^14^

Compared to the two previously published case series, our mean follow‐up duration was longer (mean (SD) of 2.2(1.6) years) compared to 6 months follow‐up within the Kaur et al. series and 12 months within the Daly et al. paper.^13^, ^14^ We do acknowledge that the SD of our follow‐up is large; however, this reflects the retrospective nature of this study and rolling onboarding to HCL systems which occurs within clinical practice in England.

As a large tertiary referral centre we offer a large selection of different HCL systems and a high degree of support and experience to individuals when onboarding. Participants within our study were offered one of five different HCL systems with the final decision driven by a combination of individual preference and MDT experience. The majority (47.1%) started on the Medtronic 780G with SmartGuard, which is in keeping with the two previous cases series in which all but one individual was started on a Medtronic system with SmartGuard. However, we do uniquely report our experience of using both Tandem‐Control IQ and Omnipod 5 with SmartAdjust.

One of the major advantages of CSII over MDI is the availability of advanced bolus features such as dual wave (split) and extended bolus options. These are particularly valuable for individuals with gastroparesis, where the unpredictability of nausea, vomiting and food absorption is common. Among the commercially available HCL systems currently available within the UK, only Tandem Control‐IQ™ and CamAPS FX algorithms support different extended/split bolus strategies during automation. Control‐IQ™ allows an extended bolus (up to 2 h) in automated mode using a split/extended strategy while the CamAPS FX has a “slowly absorbed meal” function which delivers additional insulin every 30 min for the 3–4 h following a meal in response to rising glucose levels.^21^ The perceived loss of control over insulin administration while in HCL mode appears to be a significant barrier to some individuals considering HCL therapy and was documented as a reason for declining upgrading from open loop to HCL by individuals in the non‐HCL cohort. Interestingly, only 35% of our cohort were onboarded to either Tandem Control‐IQ™ and CamAPS FX algorithms but unfortunately, data about whether these individuals were utilising the different extended/split bolus strategies wasn't recorded. This study was not powered to determine the superiority of one HCL system over another and further research is needed to determine whether HCL algorithms which support different bolus functionality demonstrate superior glycaemic outcomes in individuals with gastroparesis.

A trend towards increased carbohydrate announcement was observed within the HCL cohort. Baseline data about daily announced carbohydrate intake was only available for 8/17 individuals which may have limited our power to detect a significant difference post HCL initiation. However, the trend towards increased input of carbohydrate estimates by the pump users likely reflects increased confidence in the HCL system to manage postprandial glucose excursions effectively, without fear of delayed hypoglycaemia as individuals are more accurately announcing carbohydrates consumed.

Despite the observed trend towards increased carbohydrate announcement and a higher TDD of insulin, alongside a statistically significant reduction in HbA_1c_, there was no significant change in BMI from baseline to latest follow up. This deviates from previous HCL follow up studies which report an average weight increase of 2.2 kg, and a BMI increase of 0.7 kg/m^2^.^22^ The reason for this remains unclear but may reflect a short follow up period post HCL within individuals who had a low BMI at baseline, or it could suggest that people with gastroparesis on HCL systems gain less weight than people without gastroparesis. BMI is a crude measure of nutritional status and doesn't take into consideration vitamin status or body composition. Counterintuitively, studies have identified that a large proportion of patients with gastroparesis can be obese but have poor dietary intake and therefore BMI needs to be interpreted with caution within this cohort.^23^

This is a retrospective cohort study and therefore there are limitations to the conclusions that can be drawn. While there were attempts to assess potential benefit in gastroparesis related symptoms such as nausea, vomiting or bloating this was not uniformly captured within the electronic health records. Previous studies have been conflicting about whether symptoms of gastroparesis improve with tightening of glycaemic control. However, the follow up study on the DCCT cohort performed by the NIDDK Gastroparesis Clinical Research Consortium identified a clinically significant improvement in gastroparesis symptom score.^24^ There is a case series of three patients started on HCL who demonstrated a reduction in their Gastroparesis Cardinal Syndrome Index (GCSI) score, with one patient demonstrating a reduction in their gastric emptying half time from 422 to 185 min.^25^ However, evidence that HCL improves symptoms of gastroparesis remains limited.

We acknowledge that the cohort initiated on HCL represents a pre‐selected group of individuals deemed by the diabetes MDT to be competent to manage a HCL system. This is reflected by the observation that all but two of the HCL group were using CSII in open loop prior to transitioning to HCL. These individuals were identified as being low risk to switch to HCL and required minimal support from the diabetes MDT compared to CSII naïve individuals. It is also possible that individuals on HCL systems are highly motivated individuals and this may have introduced a degree of bias to the results obtained. However, this should not overshadow the reality that these individuals remain clinically complex, with multiple diabetes complications and a relatively high baseline HbA_1c_. Their baseline characteristics are comparable to the non‐HCL cohort in terms of HbA_1c_, TIR, diabetes duration, duration of gastroparesis, age and sex.

One limitation of this study was heterogeneity in the diagnosis of gastroparesis. Traditionally the term diabetic gastroparesis was used to describe a syndrome characterised by delayed gastric emptying in the absence of a gastric outlet obstruction. However, it is now recognised that many patients with diabetes mellitus may have a constellation of upper gastrointestinal symptoms with a normal gastric emptying study and conversely they may have evidence of delayed gastric emptying which may be asymptomatic or associated with only mild symptoms.^26^ Often these symptoms can originate from the ‘distal’ gastrointestinal tract rather than a primary gastric disorder.^27^ Differentiating gastroparesis from functional dyspepsia and other upper GI pathologies can be clinically challenging. The gold standard diagnostic test is scintigraphy based gastric emptying studies (GES) with involves ingestion of a low fat‐nuclear labelled egg‐white meal with assessment of solid emptying at 0, 1, 2 and 4 h, however reporting remains unstandardised.^28^, ^29^ Furthermore, normoglycaemia is required during testing to avoid the slow gastric emptying which is associated with both hypo and hyperglycaemia.^30^ Given the duration of gastroparesis in our cohort (mean of 10.5 years), the availability of diagnostic study reports was a particular challenge (including those performed in other institutions). We were only able to locate the results of 21 gastric emptying studies and within this the reporting of results showed a wide variation with some reporting time to 50% retention, some 1 h % retention and others just reported as “slow gastric emptying”.

Lastly, while we demonstrated improved TIR and HbA_1c_ with use of HCL we must acknowledge that all but three individuals changed brand of CGM sensor on starting HCL, which may have led to bias in improved TIR as studies have shown some differences in glucose profiles between different CGM systems used within the same individual.^31^

In summary, this retrospective observational study builds on previous small case studies demonstrating the safety and efficacy of HCL systems in individuals with type 1 diabetes and gastroparesis. Large prospective randomised controlled trials are required to investigate potential improvement in gastroparesis symptoms with HCL use and to determine potential superiority of one HCL algorithm over another.

## AUTHOR CONTRIBUTIONS

R.T and M.S drafted the manuscript; R.T, A.A., A. Sh, B.S., B.H. and M.S. were responsible for data analyses; G.G., O.G. and M.W. provided input to the study design; G.G., B.S., A.A, A. Sh, J.C. and A.S. collected data; provided R.T and M.S collected CGM data and supported statistical analysis; All authors had full access to all the data in the study, reviewed the final version of the manuscript and had final responsibility for the decision to submit for publication. M.S. and R.T. are the guarantors of this work and, as such, had full access to all the data in the study and takes responsibility for the integrity of the data and the accuracy of the data analysis.

## FUNDING INFORMATION

R.T's salary was funded through a National Institute of Health Research (NIHR) Clinical Lectureship (CL‐2023‐17‐001) and M.S's salary was funded through a National Institute of Health Research (NIHR) Clinician Scientist Fellowship award (CS‐2017‐17‐023).

## CONFLICT OF INTEREST STATEMENT

The authors declare no conflicts of interest.

## Data Availability

The data that support the findings of this study are available on request from the corresponding author. The data are not publicly available due to privacy or ethical restrictions.
